# MIR21 Drives Resistance to Heat Shock Protein 90 Inhibition in Cholangiocarcinoma

**DOI:** 10.1053/j.gastro.2017.10.043

**Published:** 2018-03

**Authors:** Andrea Lampis, Pietro Carotenuto, Georgios Vlachogiannis, Luciano Cascione, Somaieh Hedayat, Rosemary Burke, Paul Clarke, Else Bosma, Michele Simbolo, Aldo Scarpa, Sijia Yu, Rebecca Cole, Elizabeth Smyth, Javier Fernández Mateos, Ruwaida Begum, Blanka Hezelova, Zakaria Eltahir, Andrew Wotherspoon, Nicos Fotiadis, Maria Antonietta Bali, Chirag Nepal, Khurum Khan, Mark Stubbs, Jens C. Hahne, Pierluigi Gasparini, Vincenza Guzzardo, Carlo M. Croce, Suzanne Eccles, Matteo Fassan, David Cunningham, Jesper B. Andersen, Paul Workman, Nicola Valeri, Chiara Braconi

**Affiliations:** 1 The Institute of Cancer Research, London, UK; 2 Bioinformatics Core Unit, Institute of Oncology Research, Bellinzona, Switzerland; 3 ARC-Net Research Centre and Department of Pathology and Diagnostics, University of Verona, Verona, Italy; 4 The Royal Marsden NHS Trust, London and Surrey, UK; 5 Biotech Research and Innovation Centre, Department of Health and Medical Sciences, University of Copenhagen, Copenhagen, Denmark; 6 Ohio State University, Columbus, Ohio, USA; 7 Department of Medicine, University of Padua, Padua, Italy

**Keywords:** Organoid, AUY922, Bile Duct Cancer, DNAJB5, CCA, cholangiocarcinoma, DMSO, dimethyl sulfoxide, FFPE, formalin-fixed paraffin-embedded, FGFR, fibroblast growth factor receptor, GI, growth inhibition, HSP, heat shock protein, HTS, high-throughput screen, iCCA, intrahepatic cholantiocarcinoma, miRNA, microRNA, PDO, patient-derived organoid, WT, wild type

## Abstract

**Background & Aims:**

Cholangiocarcinomas (CCA) are resistant to chemotherapy, so new therapeutic agents are needed. We performed a screen to identify small-molecule compounds that are active against CCAs. Levels of microRNA 21 (MIR21 or miRNA21) are increased in CCAs. We investigated whether miRNA21 mediates resistance of CCA cells and organoids to HSP90 inhibitors.

**Methods:**

We performed a high-throughput screen of 484 small-molecule compounds to identify those that reduced viability of 6 human CCA cell lines. We tested the effects of HSP90 inhibitors on cells with disruption of the *MIR21* gene, cells incubated with MIR21 inhibitors, and stable cell lines with inducible expression of MIR21. We obtained CCA biopsies from patients, cultured them as organoids (patient-derived organoids). We assessed their architecture, mutation and gene expression patterns, response to compounds in culture, and when grown as subcutaneous xenograft tumors in mice.

**Results:**

Cells with *IDH1* and *PBRM1* mutations had the highest level of sensitivity to histone deacetylase inhibitors. HSP90 inhibitors were effective in all cell lines, irrespective of mutations. Sensitivity of cells to HSP90 inhibitors correlated inversely with baseline level of MIR21. Disruption of MIR21 increased cell sensitivity to HSP90 inhibitors. CCA cells that expressed transgenic MIR21 were more resistant to HSP90 inhibitors than cells transfected with control vectors; inactivation of MIR21 in these cells restored sensitivity to these agents. MIR21 was shown to target the DnaJ heat shock protein family (Hsp40) member B5 (DNAJB5). Transgenic expression of DNAJB5 in CCA cells that overexpressed MIR21 re-sensitized them to HSP90 inhibitors. Sensitivity of patient-derived organoids to HSP90 inhibitors, in culture and when grown as xenograft tumors in mice, depended on expression of miRNA21.

**Conclusions:**

miRNA21 appears to mediate resistance of CCA cells to HSP90 inhibitors by reducing levels of DNAJB5. HSP90 inhibitors might be developed for the treatment of CCA and miRNA21 might be a marker of sensitivity to these agents.

**Editor's Notes**

**Background and Context**

Cholangiocarcinoma is a deadly disease which is often diagnosed at an advanced stage where treatment options are limited. Novel therapies are urgently needed to improve the survival of cholangiocarcinoma patients.

**New Findings**

HSP90 inhibitors may be promising in the treatment of cholangiocarcinoma that do not express high levels of microRNA21. Mini-tumors can be grown in the lab starting from the biopsy of metastatic cholangiocarcinoma and can be used as a novel disease model for the study of cancer biology and drug sensitivity.

**Limitations**

Needs clinical validation in patients.

**Impact**

microRNAs and organoids should be included in drug discovery programmes that can identify novel therapeutic strategies for cholangiocarcinoma patients.

Cholangiocarcinomas (CCA) are tumors with dismal prognosis.^1^, ^2^, ^3^, ^4^ Surgery is the only curative treatment modality in CCA; however, less than 30% of patients are diagnosed with resectable disease.^5^, ^6^ In advanced CCA, the efficacy of systemic treatment is limited by drug resistance.^5^ A combination treatment with cisplatin and gemcitabine is recommended as first-line standard for patients with inoperable CCAs, based on data from the ABC-02 trial.^7^, ^8^, ^9^ However, long-term outcome is still poor,^5^ highlighting the need for the identification of novel therapeutics along with appropriate strategies for clinical implementation.

Attempts to test the efficacy of targeted therapies and small molecules against CCAs have been made without a proper phase of target selection and validation, leading to repeated failures in small and unselected populations of CCA patients.^10^, ^11^, ^12^, ^13^ Notably, a phase III trial failed to show a benefit from the addition of erlotinib to a gemcitabine-platinum combination in metastatic CCAs that were not enriched for the appropriate molecular subtype.^14^

Molecularly targeted small-molecule drugs are low-molecular-weight compounds that regulate biological processes and can rapidly diffuse across cell membranes so that they can reach intracellular sites of action.^15^ Small molecules have entered clinical practice for the treatment of other forms of solid malignancies, where the dependence of the cancer on specific pathways is understood. Here, we report data from a high-throughput screen (HTS) of a library of small-molecule drugs and chemical tools in human CCA cell lines that have been genetically characterized for the most frequent mutations observed in human CCA, along with validation in ex vivo and in vivo models of promising compounds and relative biomarkers of response. Our approach has enabled us to identify molecularly targeted small molecules that have activity against CCAs and related biomarkers that may inform future clinical trial design.

## Experimental Procedures

### HTS

A custom compound library including 484 small molecules was developed in the Cancer Research UK Cancer Therapeutics Unit at the Institute of Cancer Research (Supplementary Table 1). Cells were plated into a polypropylene 384-well assay plate (Greiner Bio-One, Frickenhausen, Germany) for 48 hours before compounds were screened at the final concentration of 80 nmol/L, 200 nmol/L, and 800 nmol/L in 0.3% (v/v) dimethyl sulfoxide (DMSO) by dispensing 125 nL compound solution from a source plate containing the compounds at a concentration of 32 μmol/L, 80 μmol/L, and 320 μmol/L in 2% (v/v) DMSO, into the central 320 wells of a 384-plate. 0.3% (v/v). DMSO was used as a vehicle control. Cell viability was assessed after 72 hours by fluorimetric assay (CellTiter-Blue; Promega Madison, WI). The cell viability measurement from each hit was normalized to those of cells exposed to vehicle only. Each cell line was tested in triplicate. Statistical significance *(P* < .05) was determined by 2-sided *t*-test across 3 replicates.

### Statistical Analyses

Statistical analyses were performed by GraphPad Prism 6 (La Jolla, CA). Results are expressed as mean ± SD, unless indicated otherwise. Groups that were normally distributed were compared with either a 2-tailed Student’s *t* test (for analysis of 2 groups) or using 2-way ANOVA to compare multiple groups. Non-parametric data were analyzed using a Wilcoxon–Mann-Whitney *U* test when comparing 2 groups. Significance was accepted when *P* was <.05.

### Patient-derived Organoids (PDO)

One core biopsy was obtained from a patient with advanced intrahepatic CCA (iCCA) after ethical approval within the CCR3689 protocol at the Royal Marsden Hospital (London and Surrey, UK). For the colorectal cancer PDOs, 1 core biopsy was obtained from a liver metastasis of a chemo-refractory colorectal cancer patient (protocol CCR4164). The biopsy was minced, conditioned in phosphate-buffered saline/EDTA 5 mmol/L for 15 minutes at room temperature, and digested in phosphate-buffered saline/EDTA containing 2x TrypLe (Thermo Fisher Scientific, Waltham, MA) for 1 hour at 37°C. Following digestion, mechanical force was applied to facilitate cell release in solution. Dissociated cells were collected in Advanced Dulbecco’s modified Eagle medium/F12 (Thermo Fisher Scientific), suspended in growth factor reduced matrigel (Corning Inc, Corning, NY), and seeded. The matrigel was then solidified and overlaid with 500 μL of complete human organoid medium, which was subsequently refreshed every 2 days. PDOs were cultured in Advanced Dulbecco’s modified Eagle medium/F12, supplemented with 1x B27 additive and 1x N2 additive (Thermo Fisher Scientific), 0.01% bovine serum albumin, 2 mmol/L L-glutamine, 100 units/mL penicillin-streptomycin, and containing the following additives: epidermal growth factor, noggin, R-spondin 1, gastrin, fibroblast growth factor-10, fibroblast growth factor F-basic, Wnt-3A, prostaglandin E2, Y-27632, nicotinamide, A83-01, SB202190, and hepatocytes growth factor (Pepro-Tech, London, UK). Passaging of PDOs was performed using TrypLe. PDOs were biobanked in fetal bovine serum (Thermo Fisher Scientific) containing 10% DMSO (Sigma-Aldrich, St. Louis, MO).

### PDO Histology

PDOs were harvested out of matrigel by inoculating them with 1 mL Cell Recovery Solution (Corning Inc) for 60 minutes at 4°C. Organoids were then collected in cold phosphate-buffered saline, pelleted, and fixed in formalin 10% (Sigma-Aldrich) for 60 minutes. Following fixation, organoids were washed and resuspended in 200 μL of warm agarose 2%. The agarose pellet was dehydrated using ethanol and embedded in paraffin using a standard histologic protocol.

### PDO NanoString Analysis

One hundred ng of total RNA extracted from PDOs and matching formalin-fixed paraffin-embedded (FFPE) biopsies were run with the nCounter PanCancer Progression panel (Nanostring Technologies, Seattle, WA) according to the manufacturer’s instructions. Raw data were normalized using the NanoStringNorm R package version 1.1.21 following recommended parameters and median centered by genes.

### PDO Targeting Sequencing

DNA and RNA were extracted using the Qiagen AllPrep DNA/RNA/microRNA (miRNA) Universal kit (Qiagen, Hilden, Germany). Targeted library preparation and DNA sequencing were outsourced to GATC Biotech (Constance, Germany). In brief, DNA libraries were prepared with the ClearSeq Comprehensive Cancer panel (Agilent Technologies, Santa Clara, CA) that targets 151 cancer-related genes, using SureSelectV6 chemistry (Agilent Technologies). Paired-end sequencing (2 x 125 bp) was then performed using Illumina technology.

### 3D Organoid Compound Assay and Screening

Organoids (30 μL of growth factor reduced matrigel containing 6000 cells) were seeded in 96-well cell culture plates; after matrigel solidified it was overlaid with 70 μL of complete human organoid medium. Complete medium was refreshed once after 24 hours. Compound was added 3 days later and compound-containing medium was further refreshed every 2 days. After 11 days medium was removed and replaced with 100 μL of complete human organoid medium containing 10% CellTiter-Blue Cell Viability Assay (Promega). The organoid compound screen was conducted in 96-well cell culture plates using a custom-made library of 55 compounds and 5 DMSO controls; it was conducted in triplicate, using a concentration of 1 μmol/L for all compounds.

### PDO-derived Xenografts

All in vivo experiments were performed in accordance with the local ethical review panel, the UK Home Office Animals (Scientific Procedures) Act 1986, the United Kingdom National Cancer Research Institute guidelines for the welfare of animals in cancer research,^13^ and the ARRIVE guidelines. Further details about animal experiments and additional methods can be found in the Supplementary information. Animals were housed in specific pathogen-free rooms in autoclaved, aseptic micro isolator cages with a maximum of 5 animals per cage. Food and water were provided ad libitum. One hundred μL of matrigel containing approximately 20,000 small MIR21 TRIPZ organoids were injected subcutaneously in the flank of 6- to 7-week-old NOD *scid* gamma animals (Charles River Laboratories, Wilmington, MA) while they were kept on doxycycline diet (LabDiet 5053 w/1250 ppm doxycycline blue; LabDiet, St. Louis, MO). About 10 weeks post inoculation, tumors were passaged and equal fragments of tumors were implanted subcutaneously into a next generation of mice to obtain a total of 18 mice. Eight mice were treated with vehicle, while 10 mice were treated with AUY922 (25 mg/kg intraperitoneally) 3 times a week. After 2 weeks, mice were randomized to stay on doxycycline diet or to move onto a doxycycline-free diet for an additional 2 weeks while treatment was continued. Tumor volume was determined using the following formula: =4.19*(diam1 / 4 + diam2 / 4) ^ˆ^3. After 4 weeks of treatment, mice were culled and their tumors were excised, fixed in formalin, and embedded in paraffin.

## Results

### HTS With a Small-molecule Compound Library Identified Vulnerabilities that can be Exploited for Novel Therapeutics in CCA

**Figure 1:**
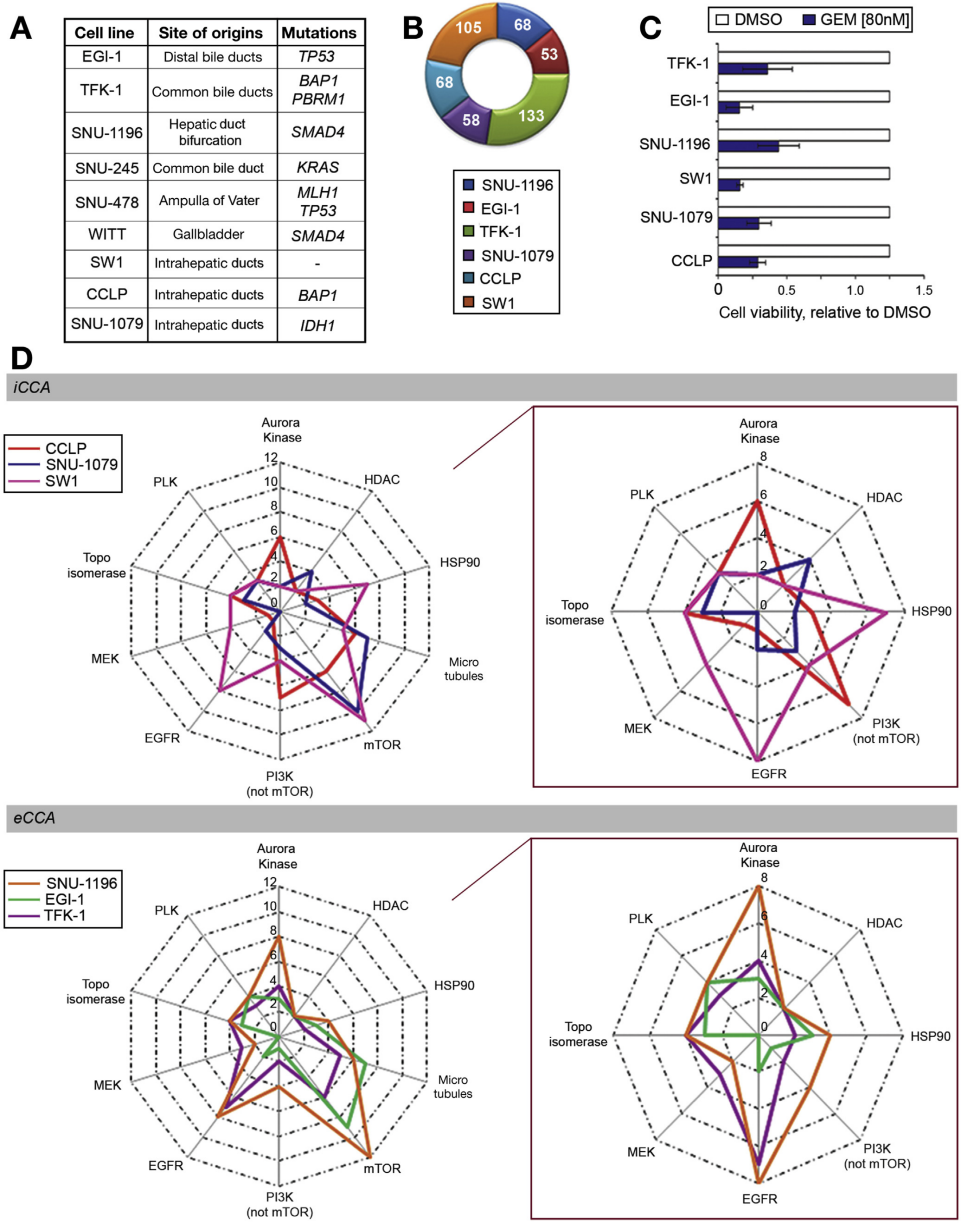
HTS using a library of small-molecule compounds in CCA cell lines. *(A)* The origin and mutational status of CCA cell lines. (*B*) HTS was performed using a custom library of 484 compounds. DMSO was used as vehicle control. Cell viability was measured by CellTiter-Blue assay and normalized to that of DMSO. HTS was run at 3 concentrations in 3 independent replicates in 6 CCA cell lines. Compounds that significantly inhibited cell proliferation compared with vehicle control *(P* <.05) at all 3 concentrations in each cell line were considered for the graph and number of compounds active per cell line is reported. (*C*) Data from the HTS relative to the activity of gemcitabine (GEM) in each cell line compared with DMSO. Bars represent mean and SD of 3 independent replicates. *P* <.05 for all cell lines. (*D*) Compounds acting on the same target were included in the same class (ie, HSP90 inhibitors). Compounds that were significantly active in comparison to DMSO *(P* <.05) at all 3 concentrations in each cell line were represented in the radar plot with the radar value representing the number of compounds per class in the selected cell line. Enrichment of selected classes of compounds was identified, such as in the case of histone-deacetylase inhibitors in SNU-1079 cells (all 4 compounds included in the library). In the callout, square data without microtubule-targeted compounds and mTOR inhibitors are shown.

To explore the activity of small molecules in CCA, we screened a library of 484 molecularly targeted small-molecule compounds (Supplementary Table 1) for their effect on the viability of human CCA cell lines. Both iCCA and extrahepatic CCA cell lines were included. Next-generation sequencing revealed that these cell lines were representative of human CCA tissues. We used a 64-gene panel that included the most frequently mutated genes in human CCA^16^ and found that mutations that are present in >10% in human tissues were represented in our cell lines, with the exceptions of *ARID1* (Figure 1*A*, Supplementary Table 2). EGI-1, TFK-1, SNU-1196, SW1, CCLP, and SNU-1079 cell lines were selected for the screening in view of their origin and their growth rate and pattern.

Compounds were screened at 3 different concentrations (80 nmol/L, 200 nmol/L, and 800 nmol/L) in triplicate for each cell line. A number of compounds (median = 68 per cell line) had a significant effect on cell viability at all concentrations tested (Supplementary Table 3, Figure 1*B*). Gemcitabine, a well-known active drug in CCA, had significant activity at all concentrations tested, in each cell line, confirming the validity of our approach (Figure 1*C*). To assess which pathway may be more relevant as a potential target of therapy in CCA, we grouped compounds with different chemical structure that acted on the same molecular target and investigated if there was an enrichment in selected molecular pathways amongst the drugs that were significantly active across the cell lines (Figure 1*D*).^17^, ^18^ We observed enrichment for microtubule-associated compounds and mTOR inhibitors in all cell lines. Clinical trials are ongoing for microtubule-targeted compounds such as Nab-Paclitaxel and mTOR inhibitors such as Everolimus. Interestingly, there was an enrichment of histone-deacetylase inhibitors among the hits in the SNU-1079 cell line, which harbors mutations in the isocitrate dehydrogenase 1 (*IDH1*) and polybromo1 (*PBRM1*) chromatin remodeling genes. In line with previous data,^19^ SNU-1079 cells also showed hypersensitivity to dasatinib (Supplementary Table 3). A number of EGFR inhibitors had a significant effect on the viability of SW1, SNU-1196, and TFK cell lines. Interestingly, both aurora kinase and heat shock protein (HSP)90 inhibitors were effective in all cell lines.

### Association Between Mutational Status of CCA Cell Lines and Their Sensitivity to Selected Compounds

**Supplementary Figure 1:**
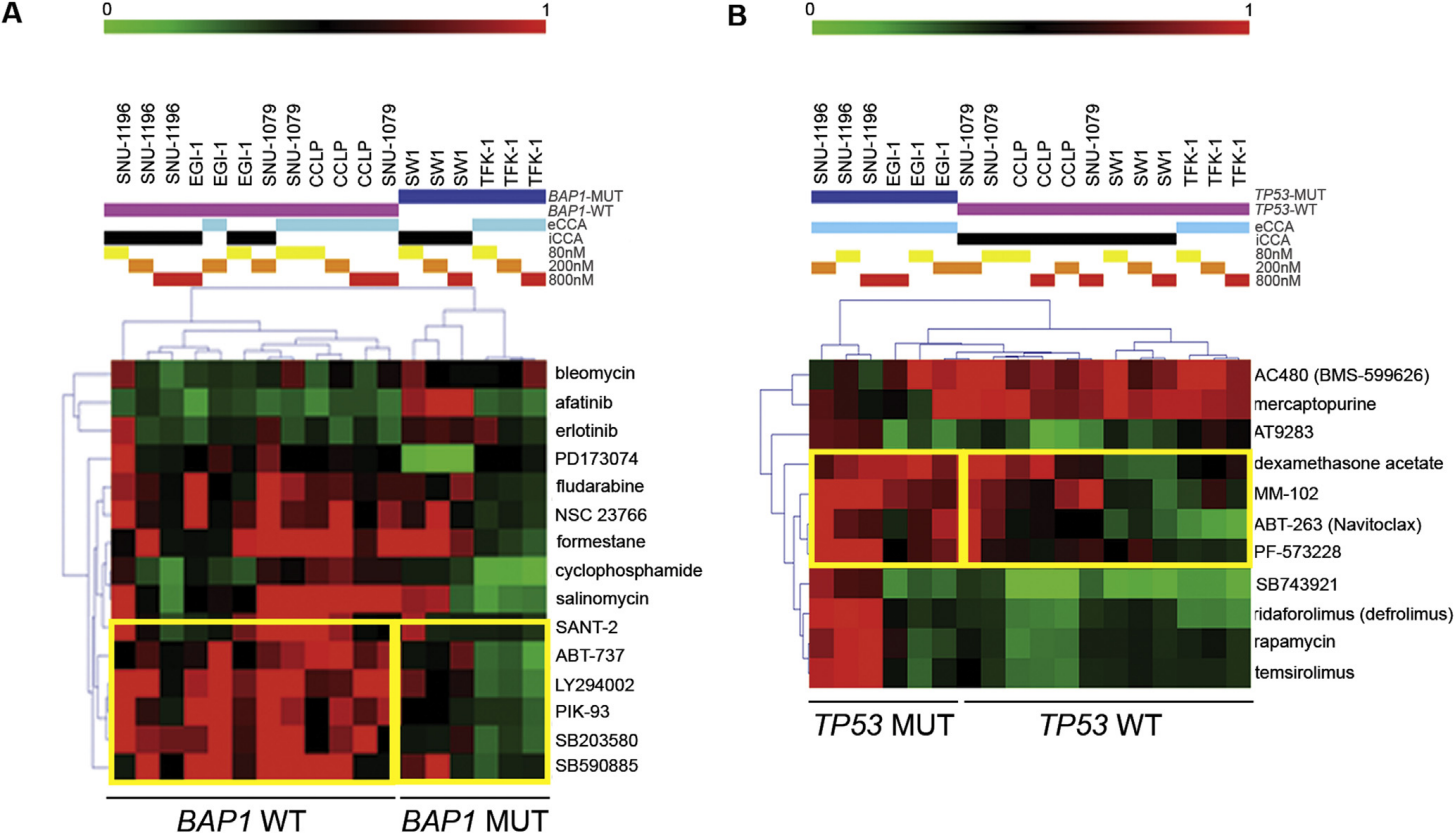
(*A&B*) Hierarchical clustering based on the sensitivity (green) or resistance (red) of cells to small-molecule inhibitors. Cells were considered sensitive if a compound reduced cell viability >20%, compared to DMSO, across 3 replicates. A Chi^2^ test was used to identify significant correlations between mutations and drug sensitivity. Mutated cells clustered together, independently of tumour origin [eCCA (light blue) vs iCCA (black)]. Of note, a concentration-response effect was observed [80nM (yellow), 200nM (orange), 800nM (red)].

To investigate whether selected mutations were associated with sensitivity to specific targeted agents, we ran an analysis for *BRCA-*associated protein (*BAP1*) and *TP53* mutations because these were present in more than 1 cell line (Supplementary Figure 1*A*,*B*). Our analysis revealed that *BAP1*-mutant (MUT) CCA cell lines were more sensitive *(P* < .05) to a range of small molecules that include compounds with activity on PI3K pathway: SANT-2 (SMO antagonist), ABT-737 (inhibitor of Bcl-(X)L, Bcl-2, and Bcl-W), LY294002 (PI3Kα/β/δ inhibitor), PIK-93 (PI3Kα/γ inhibitor), SB203580 (p38 MAPK inhibitor), and SB590885 (BRAF inhibitor). *TP53*-MUT cells did not show any increased sensitivity to the compounds we screened in comparison to WT cells. However, we noticed a significant *(P* < .05) correlation between mutations in *TP53* and resistance to PF-573228 (ATP-competitive inhibitor of FAK), ABT-263 (navitoclax, a potent inhibitor of Bcl-(X)L, Bcl-2, and Bcl-W), and MM-102 (MLLT1 inhibitor). The limited number of cell lines does not enable to draw definitive conclusions, even though these findings suggest potential associations that may deserve further investigation.

### FGFR-targeting Compounds in CCA Cell Lines

**Figure 2:**
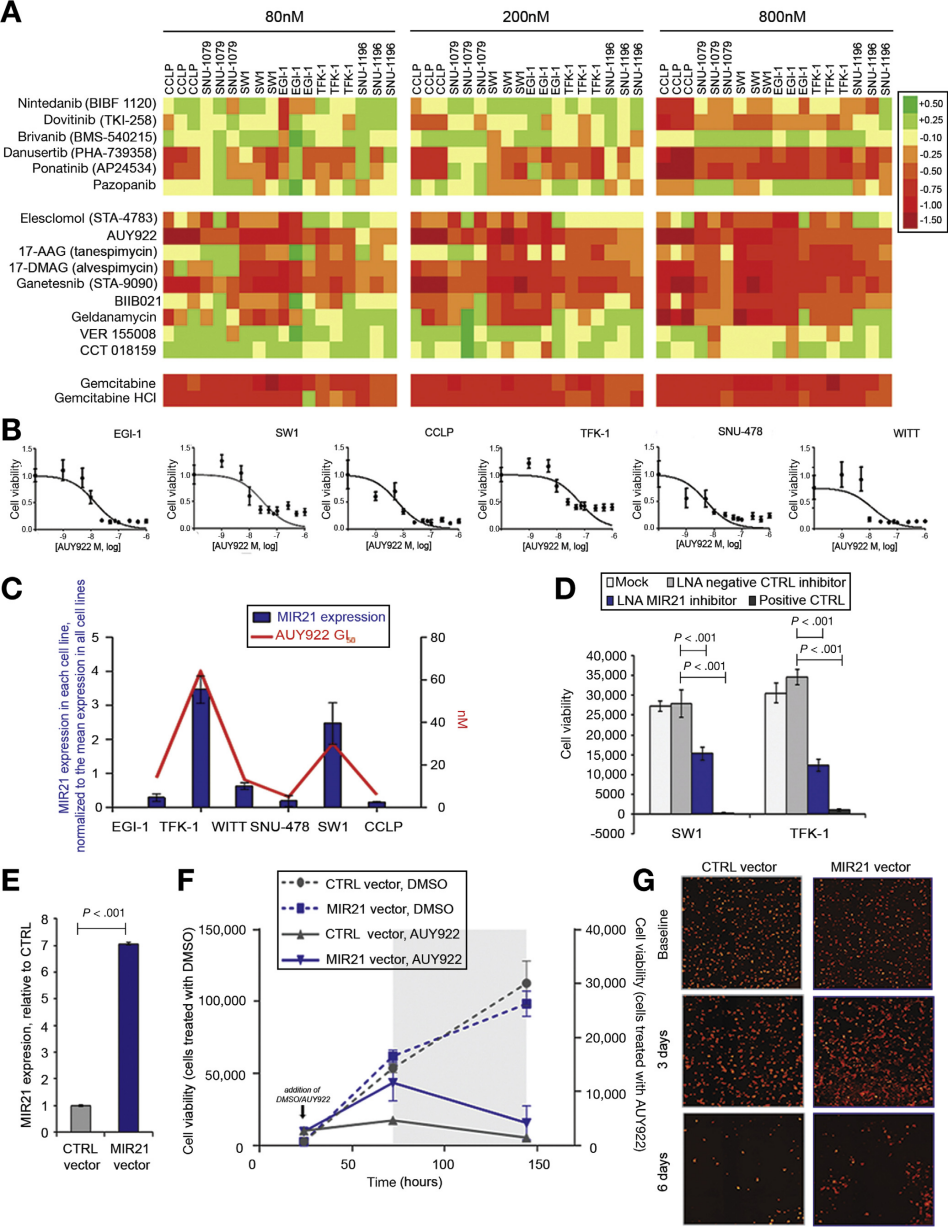
MIR21 expression is associated with sensitivity to HSP90 inhibitors. (*A*) Our compound library included 6 and 9 compounds with different degrees of activity on FGFR and HSP90. Changes in cell viability (Log scale) induced by the given compound compared with DMSO are shown. Gemcitabine is reported as positive control. (*B*) Cells were plated in 384-well plates for 48 hours and AUY922 added at scalar concentrations for 72 hours. DMSO was used as control. Cell viability was measured by CellTiter-Blue and GI_50_ generated through Prism software. (*C*) MIR21 was assessed in CCA cell lines. GI_50_ was generated by treating cells with scalar concentrations of AUY922 for 72 hours. Data represent mean of 3 replicates. (*D*) Cells were subjected to reverse transfection and plated in 96-well plates. After 48 hours, AUY922 50nmol/L was added. Cell viability was assessed by CellTiter-Blue. Positive control cell death was used as transfection control. (*E*) CCLP cells were infected with MIR21 or CTRL TRIPZ viral vector to generate stable clones. miR21 expression was assessed by Taqman assays and normalized to that of RNU48. Bars represent mean and SD of 3 replicates. (*F*) Doxycycline-induced cells were plated in 96-well plates and treated with DMSO or AUY922 (10 nmol/L). After 72 hours, doxycycline was removed to deactivate MIR21 expression (indicated by grey area). Cell viability was measured at selected time points by a Celigo S cytometer and plotted against Y axis (DMSO-treated cells toward left Y axis, while AUY922-treated cells toward right Y axis). Bars represent SD of 12 replicates. Statistical analysis is reported in Supplementary Table 4. (*G*) Representative pictures at different time points are shown.

Given emerging data on the activation of the fibroblast growth factor receptor (FGFR) pathway in CCA,^20^, ^21^, ^22^, ^23^ we looked at the effect on cell viability of the 6 compounds in our screen that act on FGFR. The effect of these compounds on cell viability was most consistent at the highest concentration tested, 800 nmol/L (Figure 2*A*). Whilst brivanib (VEGFR/FGFR inhibitor) and the multi-kinase inhibitor pazopanib had no effect, both danusertib (a pan-aurora kinase inhibitor with an off-target effect on FGFR1) and ponatinib (a Src and Bcr-Abl kinase inhibitor with activity on all 4 FGFRs)^24^ reduced CCA cell viability. However, we acknowledge that our system may not be ideal for the assessment of angiogenesis/stroma-directed drugs and that our cells are not known to carry FGFR2 alterations.

### HSP90 Inhibitors are Effective in CCA Cell Lines

We have previously observed enrichment in aurora kinase and HSP90 inhibitors amongst the significant hits from our CCA HTS. While aurora kinase inhibitors appear to be quite toxic in solid tumours,^25^ a recent report showed that HSP90 inhibition is effective and tolerable in in vivo CCA preclinical models.^26^ HSP90 inhibition is attractive in CCA because HSP90 interacts with and controls a variety of client proteins that play a key role in CCA pathogenesis, such as EGFR, PTEN, PI3K, HER2, HER3, and PRKA. Moreover, recent evidence suggests that HSP90 inhibition is remarkably effective in tumors with FGFR fusions and activation of the IL6/STAT pathway.^27^, ^28^

Our small-molecule compound library included 9 HSP90 inhibitors, including those from different chemical series, and 78% were active across our CCA cell line panel, with the highest activity recorded for AUY922, 17-AAG, 17-DMAG, ganetespib, and BIIB021 (Figure 2*A*). Notably, the growth inhibitory (GI)_50_ of AUY922, a potent HSP90 inhibitor,^29^ was in the nanomolar range in all of the CCA cell lines tested (Figure 2*B*). We found no correlation between the most frequent mutations in CCA and the activity of the HSP90 inhibitors in our CCA cell lines.

### MIR21 as Driver of Resistance to HSP90 Inhibitors

**Supplementary Figure 2:**
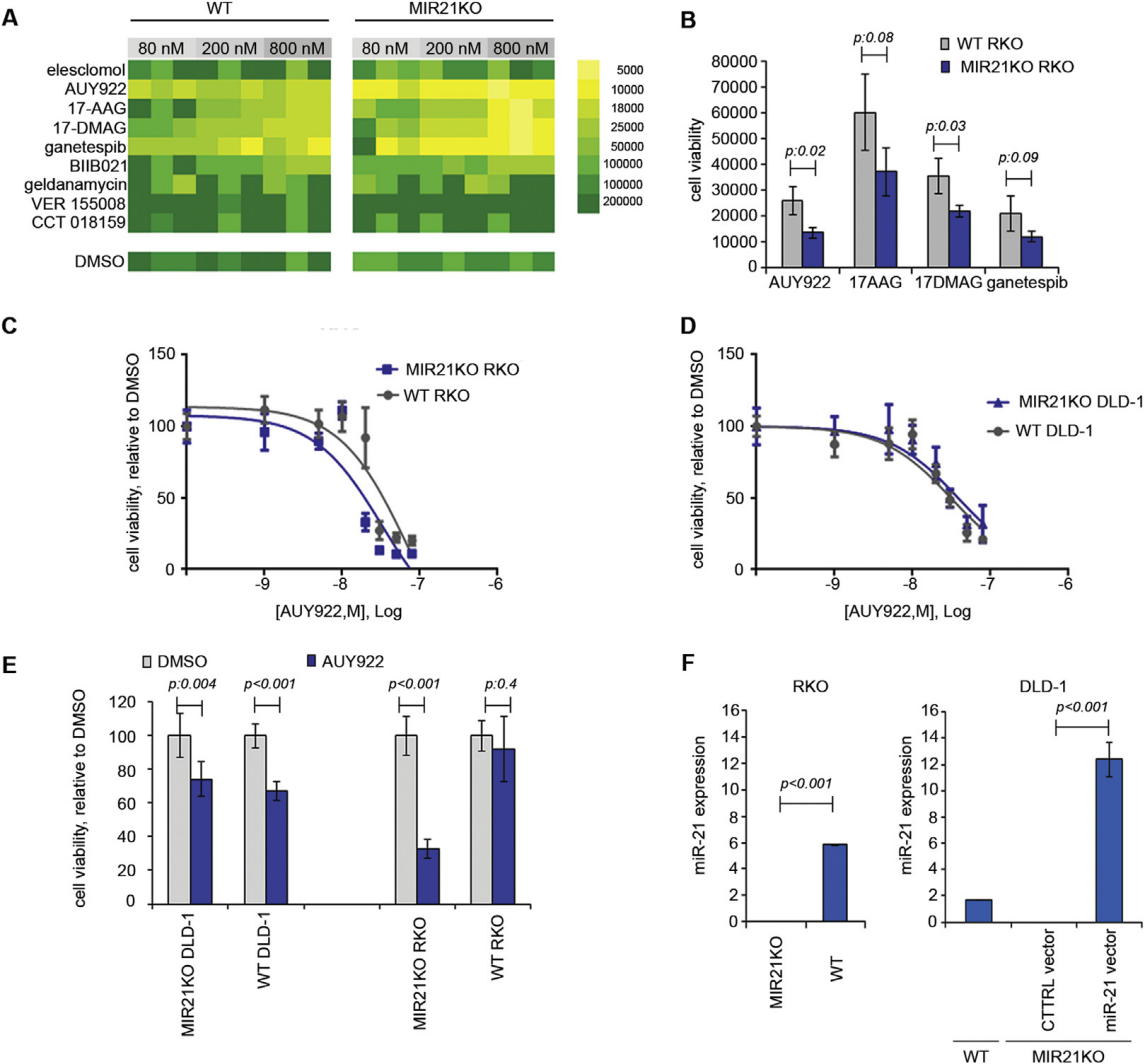
(*A&B*) Data from the compound screening in RKO cells are shown as a colour map or a bar graph (200nmol/L). Bars represent mean and standard deviation of three replicates. (*C*&*D*) Cells were plated in 96-well plates for 24 hours and AUY922 added at scalar concentrations for 72 hrs. DMSO was used as control. Cell viability was measured by CellTiter-Blue and GI_50_ generated through Prism software. Bars represent mean and SD of 6 replicates. (*E*) Cell were treated with the same concentration of AUY922 (10nmol/L) for 72 hours and cell viability assessed by CellTiter-Blue. Bars represent mean and SD of 6 replicates. (*F*) miR21 expression was assessed by Taqman assays and normalized to that of RNU48. Bars represent mean and SD of three replicates. Baseline miR-21 expression is higher in RKO compared to DLD-1 WT cells.

Previously, miRNAs have been shown to modulate drug sensitivity and to act as biomarkers of drug response.^1^, ^4^, ^30^, ^31^, ^32^, ^33^, ^34^, ^35^, ^36^, ^37^ MIR21 is an oncogenic miRNA that drives CCA pathogenesis and sensitization to conventional chemotherapy drugs.^38^, ^39^ Thus, we investigated if MIR21 could be used as a biomarker of response to HSP90 inhibition in CCA. Interestingly, we noticed that MIR21 expression reflected the sensitivity of CCA cells to AUY922, as cell lines with high levels of MIR21 expression had higher GI_50_ values for AUY922 (Figure 2*C*). Sensitivity to AUY922 was significantly increased in CCA cells transfected with a locked nucleic acid MIR21 inhibitor compared with those transfected with a negative control locked nucleic acid inhibitor (Figure 2*D*). To validate the relationship between MIR21 expression and AUY922 sensitivity, we conducted a high-throughput compound screen in RKO cells that had been engineered to knock out the *MIR21* locus (MIR21KO) and parental isogenic wild type (WT) cells.^40^ A number of HSP90 inhibitors produced a larger reduction in cell viability in MIR21KO cells in comparison with WT cells (Supplementary Figure 2*A*), with AUY922, 17-AAG, 17-DMAG, and ganetespib showing the highest activity. When treated with HSP90 inhibitors, MIR21KO RKO cells were more sensitive than WT RKO cells (Supplementary Figure 2*B*). Correspondingly, the GI_50_ for AUY922 was found to be 35 nmol/L in WT cells and 17 nmol/L in MIR21KO cells (Supplementary Figure 2*C*). Interestingly, we could detect no difference in the sensitivity to AUY922 in WT and MIR21KO DLD1 cells, which is consistent with the lower baseline level of MIR21 in DLD-1 cells and their likely lower dependence on MIR21 (Supplementary Figure 2*D*). Indeed, DLD-1 WT cells were more sensitive to AUY922 than RKO WT, while silencing of MIR21 in RKO cells restored their sensitivity (Supplementary Figure 2*E*,*F*).

**Supplementary Figure 3:**
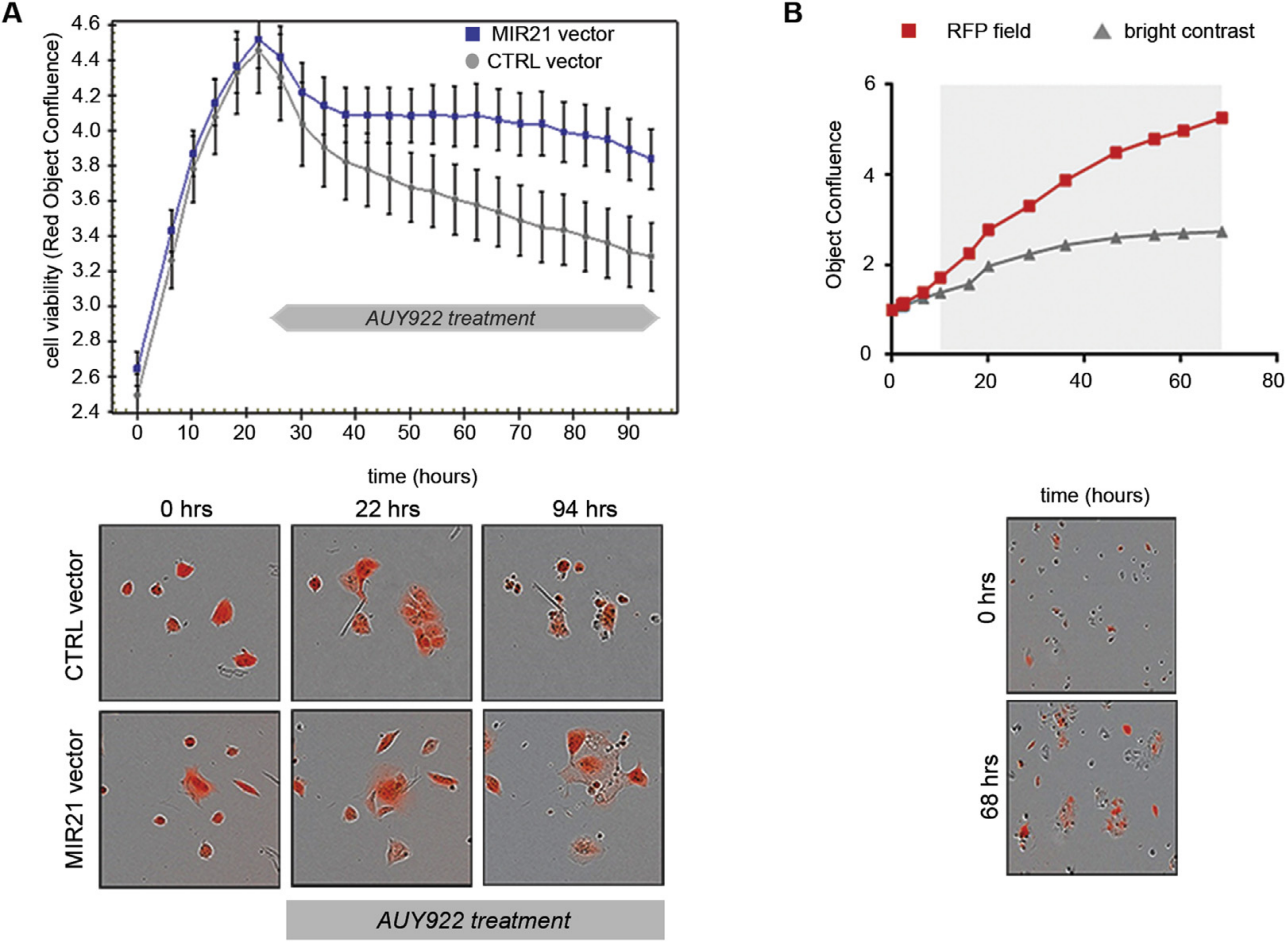
(*A*) miR-21KO DLD-1 cells were stably infected with a Tet-on TRIPZ vector enabling over-expression of miR-21 and RFP, or a control (CTRL) empty vector expressing RFP. Cells were exposed to doxycycline to induce miR-21/CTRL and RFP expression that were under the same promoter. RFP+ (red) cells represented induced infected cells. Induced cells were plated in 96-well plates and AUY922 added 22 hrs later. Cell viability and RFP confluency were monitored and measured at intervals by the Incucyte Zoom. Data represent mean and SD of 12 replicates. Difference between the two curves was statistically significant (*P* < .05). Representative images are shown for selected time points. (*B*) miR-21KO DLD1 cells were co-cultured with Tet-on miR-21KO miR-21 vector DLD-1 cells. Cells were induced, plated in 96-well plate, exposed to doxycycline, and treated with AUY922 (10nmol/L). RFP+ cells represented cells that over-expressed miR-21, while RFP- (bright-field) cells represented miR-21KO cells. Phase contrast and RFP confluency were monitored and measured at intervals by the Incucyte Zoom. Cell proliferation was reduced in RFP- cells, while RFP+ cells were able to expand and proliferate. Images are shown for selected time points.

**Supplementary Figure 4:**
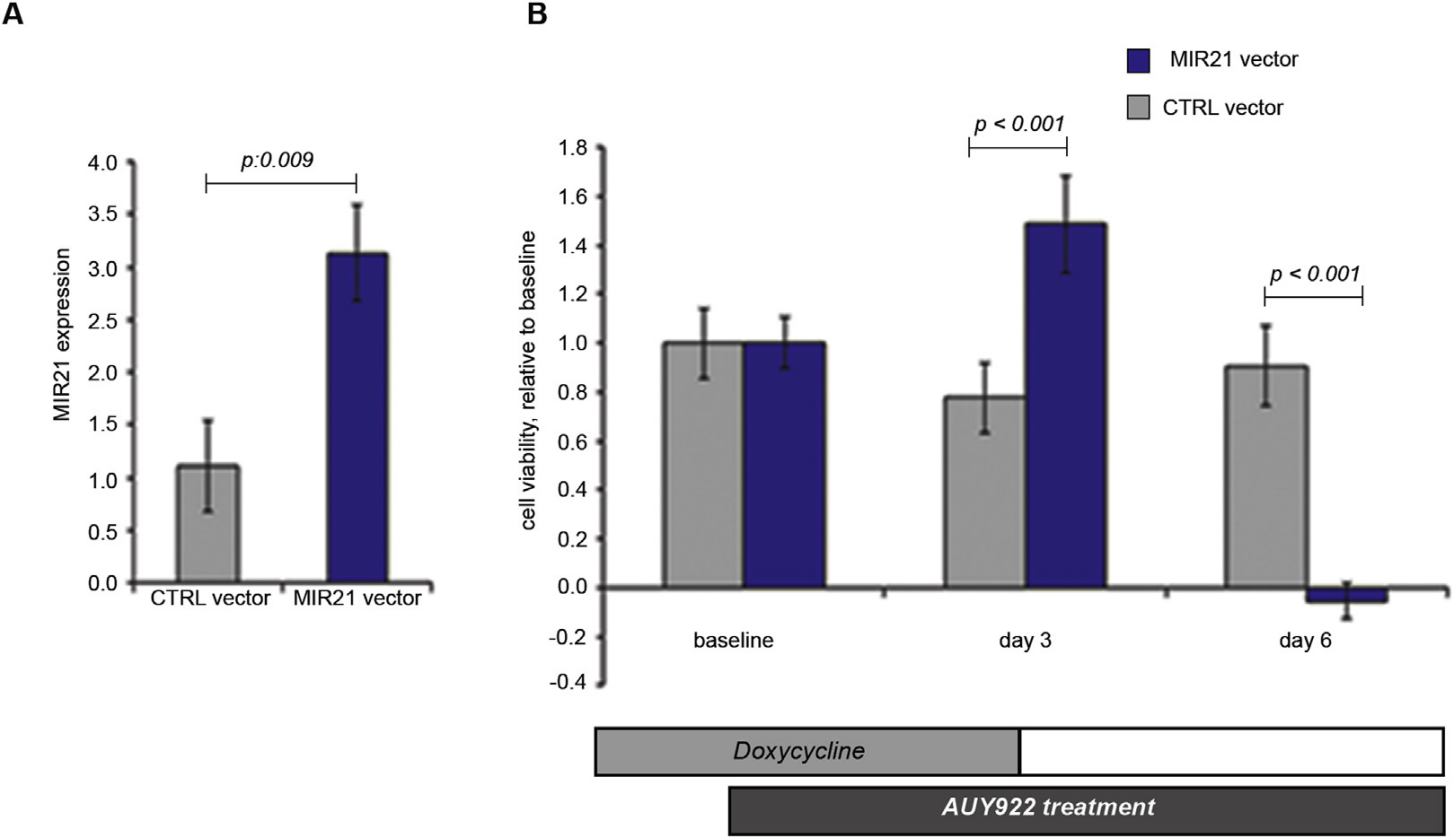
EGI-1 cells were infected with miR-21 or CTRL TRIPZ viral vector to generate inducible stable clones. (*A*) miR21 expression was assessed by Taqman assays and normalized to that of RNU48. Bars represent mean and SD of three replicates. (*B*) Doxycycline-activated cells were plated in 96 well plates and treated with sub-lethal concentrations of AUY922 (5nmol/L). After 72 hrs doxycycline was removed to deactivate miR-21 expression. Cell viability was measured at selected time points by Celigo S. Bars represent mean and SD of 12 replicates.

To validate the role of MIR21 in driving resistance to HSP90 inhibition, we infected MIR21KO DLD-1 cells with an inducible MIR21 or control (CTRL) viral vector (Supplementary Figure 2*F*) and monitored their response to AUY922. Enforced expression of MIR21 significantly increased resistance to AUY922 *(P* < .05), when compared with the effect of infection with an empty CTRL vector (Supplementary Figure 3*A* and Supplementary Video 1). Indeed, in co-culture with non-infected MIR21KO DLD-1 cells, MIR21-induced DLD-1 cells could proliferate in the presence of AUY922 (Supplementary Figure 3*B* and Supplementary Video 2). To ascertain if these results could be extended to CCA, we generated Tet-on inducible clones for the over-expression of MIR21 in the CCLP cell line (Figure 2*E*). In line with previous data, CCLP cells with enforced expression of MIR21 were significantly more resistant to AUY922 than cells transfected with the CTRL vector. Accordingly, deactivation of the Tet-on system restored sensitivity to AUY922 in CCLP cells (Figure 2*F*,*G*, Supplementary Table 4). Comparable data were also obtained in the EGI CCA cell line (Supplementary Figure 4).

### DNAJB5 is a Mediator of MIR21-dependent Resistance to AUY922

**Figure 3:**
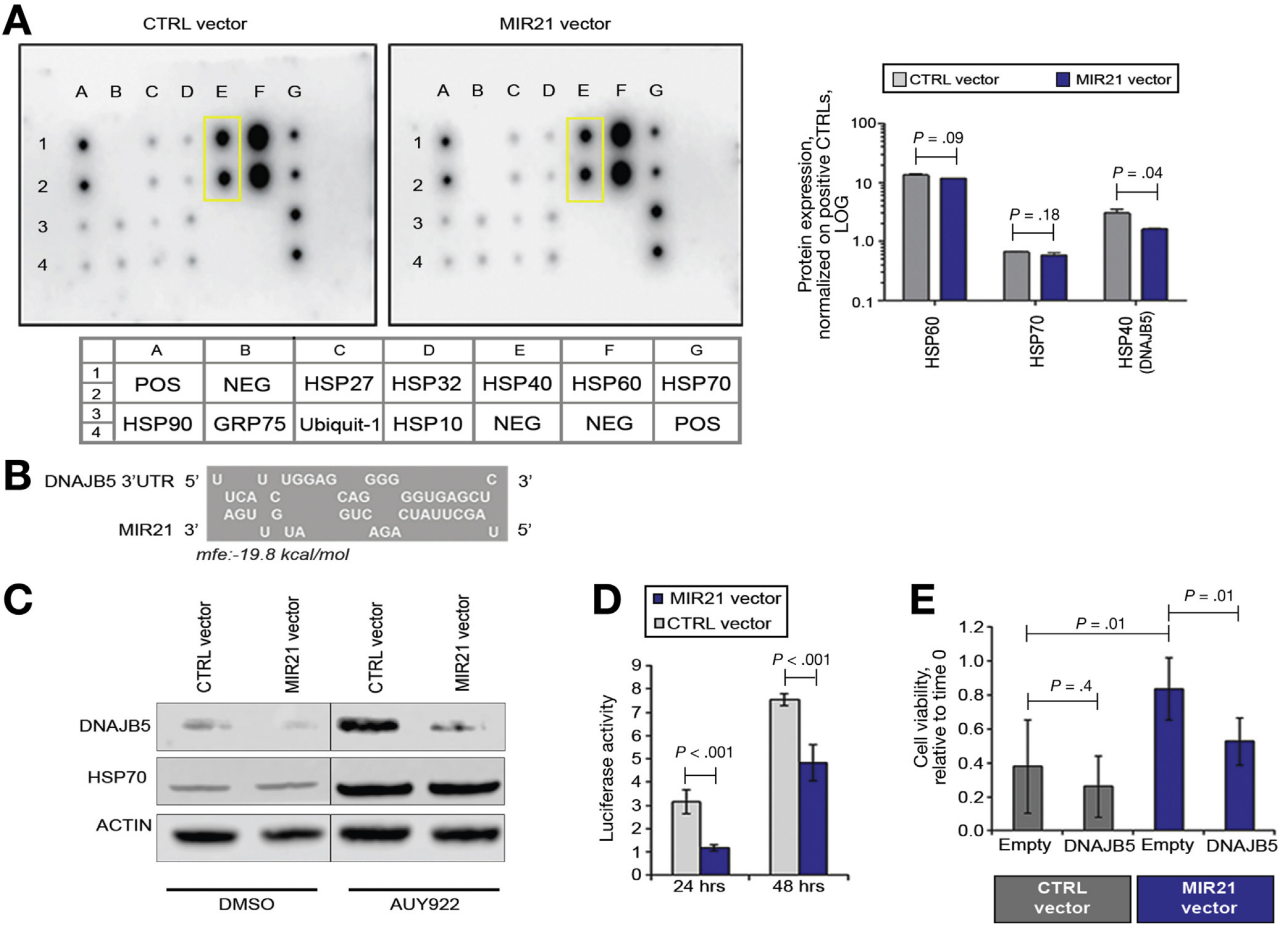
DNAJB5 is a target of MIR21. (*A*) MIR21 and CTRL TRIPZ viral vector CCLP cells were treated with AUY922 for 72 hours and proteins collected for the HSP array. Dots in the *yellow boxes* represent DNAJB5 protein expression in duplicate. Full details of the antibody plate map are provided below the blots. Quantification of protein expression normalized on the averaged positive controls is shown in the right panel. Bars represent LOG10 of mean and standard deviation of 2 replicates. Linear fold change of MIR21 vector relative to CTRL vector is 0.89 for HSP60, 0.88 for HSP70, and 0.52 for HSP40. (*B*) Schematic representation of the MIR21 binding site within the 3’UTR of DNAJB5 mRNA (RNAHybrid). (*C*) MIR21 and CTRL TRIPZ-infected CCLP cells were treated with DMSO and AU922 for 72 hours. HSP70 was used a marker of target engagement for AUY922 activity. (*D*) Cells were plated in 6-well dishes and transfected with a pMirTarget vector containing DNAJB5-3’UTR. Luciferase activity was read after 24 and 48 hours and normalized to renilla activity for each transfected well. Bars represent mean and SD of 3 replicates. (*E*) Cells were transfected with a plasmid over-expressing DNAJB5 or an empty plasmid pCMV6 for 24 hours, and then treated with AUY922. Cell viability was measured 48 hours later using CellTiter-Blue. Bars represent mean and SD of 6 replicates.

To gain insight into the relationship between MIR21 and the HSPs, we measured the expression levels of a panel of HSPs and co-chaperones in Tet-on MIR21 vector CCLP cells treated with AUY922. A multiplex sandwich immunoassay showed a reduction in the level of HSP40 (encoded by DnaJ heat shock protein family (Hsp40) member B5, *DNAJB5*) in MIR21 vector cells compared with CTRL cells (Figure 3*A*). In silico analysis of the DNAJB5 sequence revealed a binding site for MIR21 within its 3’UTR (Figure 3*B*). Western blot analysis confirmed induction of DNAJB5 upon AUY922 treatment and reduction in DNAJB5 expression in MIR21 over-expressing cells (Figure 3*C*), and a luciferase reporter assay confirmed a direct interaction between MIR21 and the 3’UTR of DNAJB5 (Figure 3*D*). Interestingly, enforced expression of DNAJB5 in MIR21 over-expressing cells re-sensitized CCLP cells to AUY922 (Figure 3*E*), confirming that DNAJB5 may be a mediator of MIR21-induced resistance.

### Correlation Between MIR21 Expression and Sensitivity to AUY922 in PDOs and PDO-derived Tumor Xenografts

**Figure 4:**
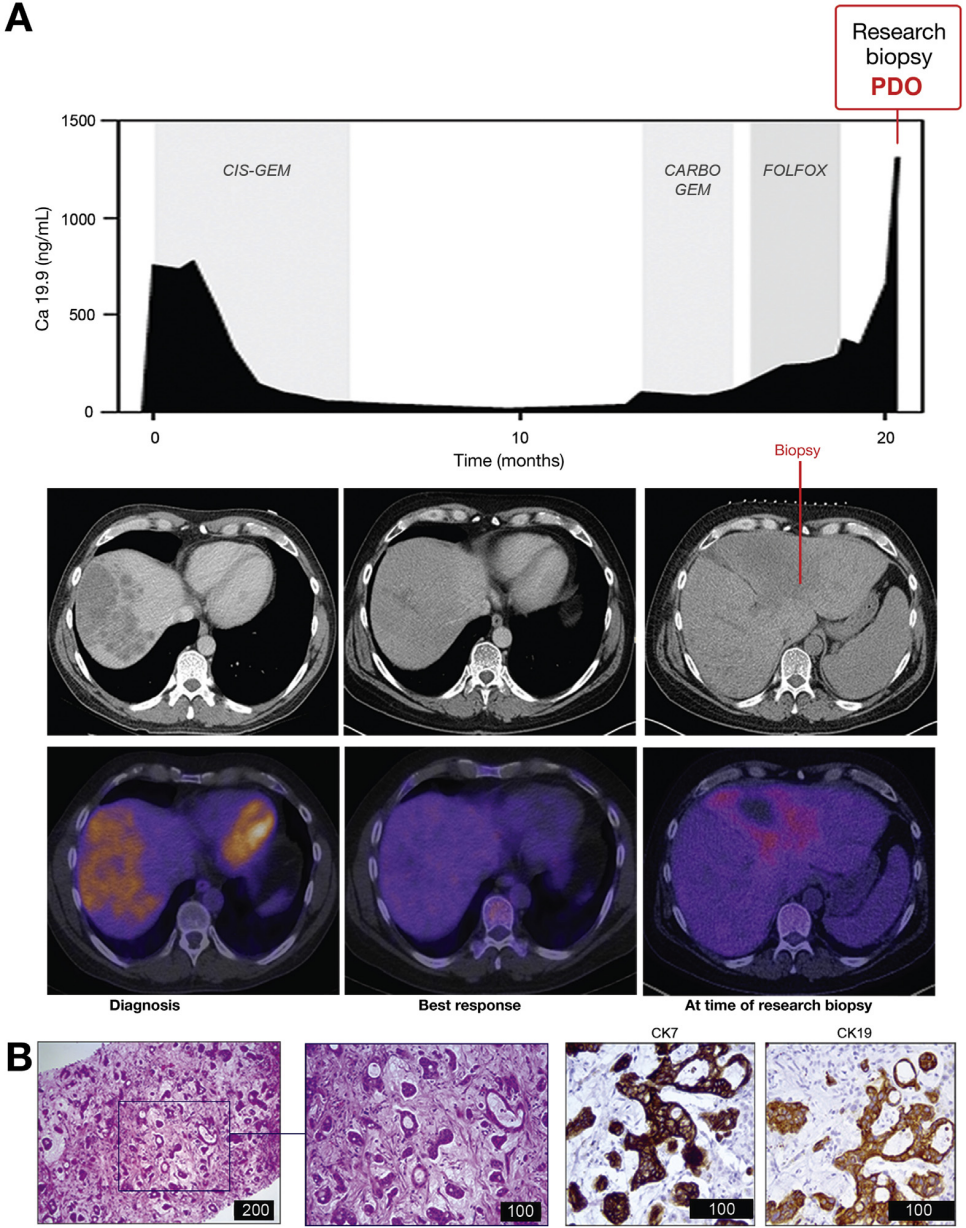
PDOs were derived from a patient with metastatic chemo-refractory iCCA. *(A)* Trend in serum Ca19.9 is represented over time. Biochemical and radiological partial response was observed to chemotherapy with cisplatin and gemcitabine, while progressive disease was recorded after carboplatin-gemcitabine or Folfox chemotherapy. Computed tomography (top panel) and positron-emission tomography (bottom panel) images are shown for indicated time points. (*B*) H&E (left) and immunohistochemistry for Cytokeratines 7 and 19 (right) of the FFPE research biopsy. Scale bars in μm.

**Figure 5:**
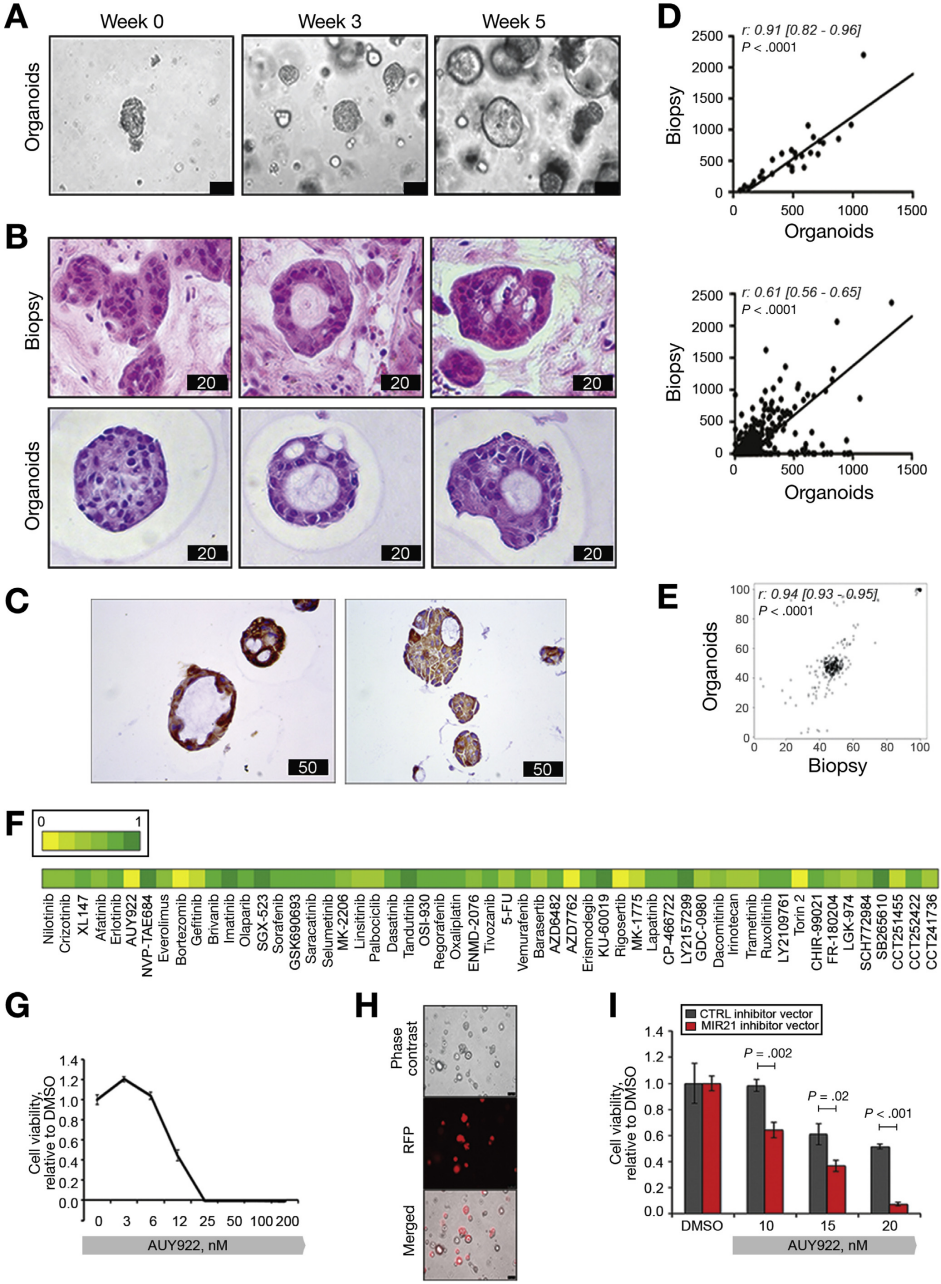
Sensitivity of CCA PDOs to HSP90 inhibition with and without MIR21 modulation. (*A*) Phase-contrast images of PDOs derived from 1 biopsy core of an iCCA. Bars indicate 100 μmol/L. (*B*) One biopsy core was embedded in paraffin, while another core was used to establish PDOs. PDOs were embedded into paraffin and stained for H&E. Bar score in μm. (*C*) IHC staining for CK7 and CK19 in PDOs. Bar score in μm. (*D*) Total RNA was extracted from the FFPE biopsy and the matching PDOs, and subjected to NanoString analysis. Correlation of gene expression is shown for housekeeping genes (top) and total gene expression (bottom). (*E*) DNA was extracted from the FFPE biopsy and the matching PDOs and subjected to targeting sequencing. Correlation between variant reads frequency is shown. (*F*). CCA PDOs were plated in 96-well plates and treated with a number of compounds (1 μmol/L) in triplicate. Cell viability was tested after 11 days with CellTiter-Blue. Mean of 3 replicates are shown relative to DMSO with DMSO set at 1. (*G*) CCA PDOs were treated with scalar concentrations of AUY922 in triplicate. (*H*) CCA PDOs were infected with a MIR21-inhibitor or control TRIPZ viral vector. RFP+ cells indicate infected cells. Scale bars indicate 100 μm. (*I*) TRIPZ infected CCA PDOs were treated with scalar concentrations of AUY922.

**Figure 6:**
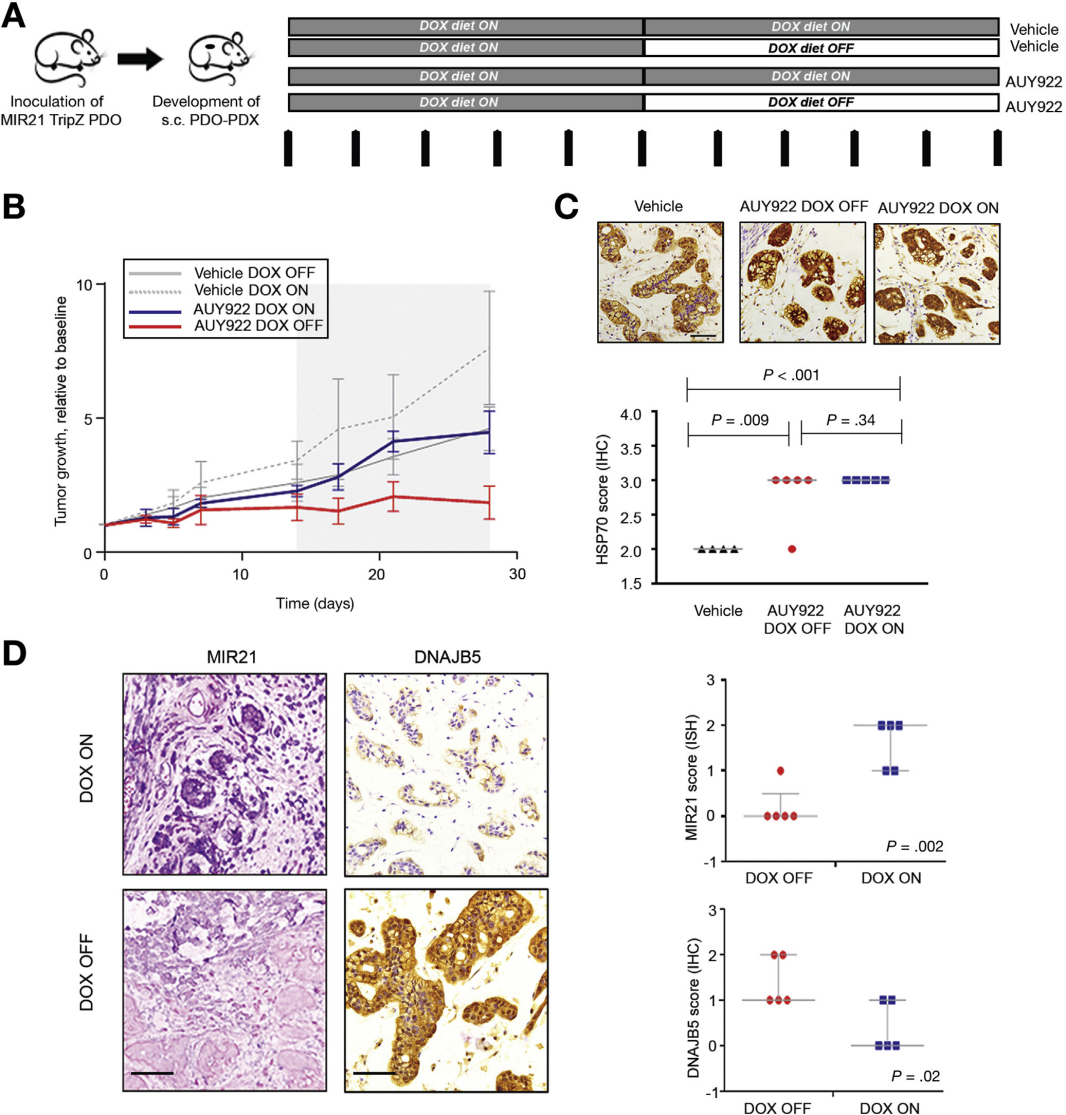
MIR21 modulation drives sensitivity to AUY922 in CCA PDO-derived animal model. (*A*) Schematic representation of in vivo studies. Vertical arrows indicate administration of DMSO or AUY922 25 mg/Kg. (*B*) Tumor growth curves across different groups. Data represent mean and standard error (n = 5 for AUY922 treated, n = 4 for DMSO treated). *P* values are shown in Supplementary Table 5. Grey area represents the period with different diets. (*C*) HSP70 staining was performed as evidence of target engagement after AUY922 exposure. As expected, there was an increase in HSP70 expression after AUY922 treatment but this was not different between the 2 randomized groups, excluding differences in animal dosing. Scale bars: 100 μm. (*D*) Withdrawal of doxycycline diet from mice was associated with a significant inactivation of MIR21 expression and over-expression of DNAJB5. Representative pictures of in situ hybridization for MIR21 and IHC for DNAJB5 are shown. Scale bars: 100 μm. On the right quantification is represented. Bars indicate median with interquartile ranges.

**Supplementary Figure 5:**
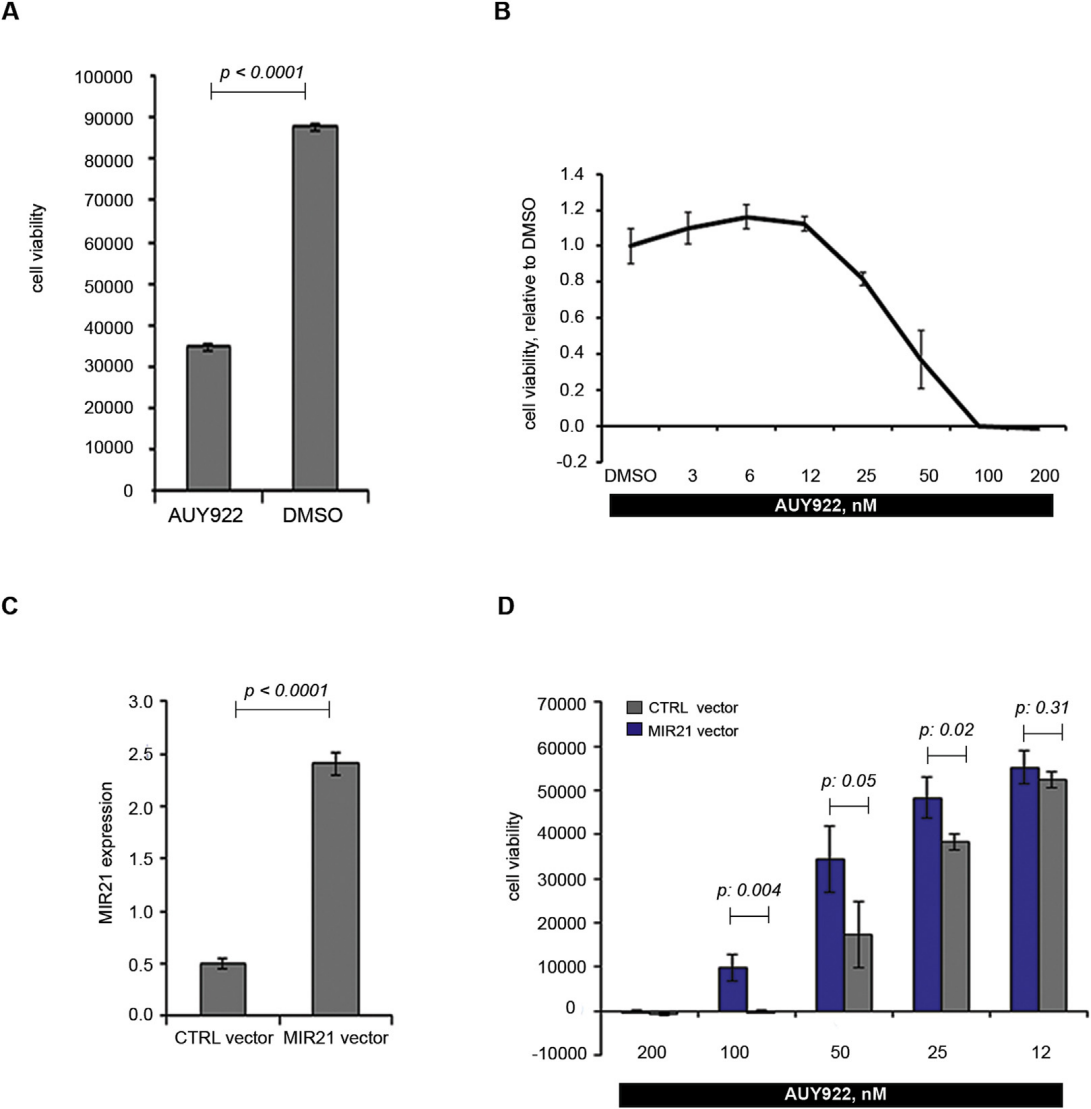
CRC PDOs were plated in 96-well plates and treated with AUY922 in triplicates at 20nmol/L (*A*) or at scalar concentrations *(B)*. (*C*) CRC PDOs were infected with a miR-21-over-expressing or control TRIPZ viral vector. (*D*) TRIPZ infected CRC PDOs were treated with scalar concentrations of AUY922. miR-21 expressing cells were more resistant to AUY922.

PDOs have recently emerged as organotypic cultures that recapitulate the complex 3-dimensional organization of cancer better than 2D tumor cell lines.^41^, ^42^, ^43^ To assess the clinical relevance of our findings, we tested AUY922 activity in PDOs established from the liver biopsy of a chemoresistant iCCA patient (Figure 4, Figure 5*A*). PDOs retained the same morphology of the primary tumor (Figure 5*B*), as well the same positivity for cytokeratin 7 and 19 (Figure 5*C*, Figure 4*B*). Gene expression profiling showed that the transcriptome of PDOs recapitulated that of the parental tissue (with a Spearman r score of 0.91 for the housekeeping genes, and 0.61 for the whole transcriptome *[P* < .0001]) (Figure 5*D*). DNA sequencing confirmed that the genetic background of the PDOs matched that of the parental biopsy, with a Spearman r score of 0.96 for SNVs (Figure 5*E*). CCA PDOs were tested against a panel of small-molecule compounds and confirmed resistance to fluorouracil and oxaliplatin that patient had received before the development of PDO (Figure 5*F*). CCA PDOs were sensitive to AUY922 (Figure 5*F*,*G*), and this sensitivity was significantly enhanced after inducible inhibition of MIR21 (Figure 5*H*,*I*). In parallel, PDOs derived from a colorectal cancer patient with low endogenous expression of MIR21 were characterized (personal data) and tested against AUY922 before and after MIR21 expression, confirming the relationship between miRNA expression and sensitivity to HSP90 inhibition (Supplementary Figure 5). Next, we generated CCA PDO-derived tumor xenografts by inoculating Tet-on MIR21 PDOs in the flank of NOD *scid* gamma mice. Mice were treated with AUY922 or vehicle while changes in their diet were applied to modulate the expression of MIR21. After 2 weeks of treatment mice were randomized to stay on doxycycline diet (DOX-ON) or changed to a doxycycline-free (DOX-OFF) diet. While a non-significant change was observed for vehicle-treated mice, AUY922-treated mice on DOX-OFF diet achieved a significantly better tumor response than animals that remained on a doxycycline diet (Figure 6*A*–*C*, Supplementary Table 5). MIR21 expression was confirmed to be inactivated in the tumor after withdrawal of doxycycline diet, while an increase in DNAJB5 protein expression was detected (Figure 6*D*).

## Discussion

The best way to treat advanced CCA is still a matter of debate within the clinical and scientific community.^5^, ^6^ The ABC-02 trial has proven the efficacy of platinum-gemcitabine combination chemotherapy in the first-line setting.^7^ However, a series of clinical trials have failed to demonstrate any benefit from targeted therapies in CCA.^11^, ^12^, ^14^, ^44^ Despite advances having been made in the genetic and molecular characterization of biliary tract cancers, none of the clinical trials were designed with an appropriate strategy of patient selection based on pre-clinical evidence. In this study, we explored the activity of a plethora of small-molecule compounds and probes that have shown activity in other cancers. In this way, we hoped to identify drug candidates and appropriate biomarkers for use in, and to aid patient selection for, future clinical trials. We propose that the decision to use targeted therapies should be based on the molecular characterization of a tumor, rather than its site of origin. Hence, in this study we included cell lines originating from all types of CCA.

As expected, we did observe significant activity for gemcitabine and compounds that impair microtubule dynamics and cause cell cycle arrest. We also found that compounds that inhibit mTOR signaling had activity in CCA cell lines, consistent with previous evidence suggesting that the mTOR pathway is involved in CCA tumorigenesis and that sirolimus may induce partial remissions in CCA patients.^45^, ^46^ Nonetheless, we did not focus on these compounds given that clinical trials are ongoing and may provide additional insights. The observation that histone-deacetylase inhibitors were enriched amongst the hits in SNU-1079 cells was in line with previous observations on the effect of *IDH* mutations on the impairment of histone demethylation.^47^

We and colleagues at The Institute of Cancer Research have an interest in the therapeutic applications of HSP90 inhibitors and biomarkers of sensitivity to these agents, and we co-discovered the highly potent and selective HSP90 inhibitor AUY922.^48^ Shirota et al^26^ have recently shown that HSP90 inhibitors have potent in vitro and in vivo anti-proliferative activity in CCA, prompting us to investigate potential biomarkers of sensitivity to HSP90 inhibition in our study. HSP90 inhibitors, including AUY922, have shown an acceptable toxicity profile in humans in phase I clinical trials,^49^, ^50^, ^51^ and are currently investigated in phase II clinical trials for lung and breast cancers. To date, no reports are available on the role of AUY922 in biliary tract cancer patients. More interestingly, growing evidence points to a role of HSP90 inhibitors in facilitating the anti-tumor activity of immune cells.^52^, ^53^ We showed that CCAs are characterized by an immuno-deregulation that creates an immunosuppressive milieu^54^; thus, HSP90 may be used to reactivate an anti-tumor response in CCA. HSP90 is a key component in a multi-chaperone complex involved in the post-translational folding of a number of client proteins, including microRNA-regulated proteins such as argonaute2 (AGO2).^55^, ^56^, ^57^ We reasoned microRNAs may be good biomarker candidates given their capacity to act on several HSP90-associated proteins that drive tumorigenesis and drug resistance. MIR21 was previously shown to modulate cytotoxic drug response^58^ and is predicted to target genes that act as client proteins for HSP90.^2^, ^3^, ^20^, ^21^, ^38^, ^59^ However, microRNAs were not studied as mediators of the response to HSP90 inhibitors to our knowledge.^60^ We observed that MIR21 can drive tumor cell proliferation in the presence of HSP90 inhibitors. Our data suggest that it would be useful to carry out further studies of the biomarker potential of MIR21 as a guide treatment with HSP90 inhibitors, as well as to pursue the combination of HSP90 inhibitors with MIR21 inhibitors in CCA. Moreover, our data suggest a generalized mechanism of resistance to HSP90 inhibition and may be applied to second-generation HSP90 inhibitors that may be clinically more attractive.^61^, ^62^ HSP70 is a well-known compensatory mechanism of HSP90 inhibition. The stress-inducible HSP70 is central in promoting protein folding. As elegantly described by Hartl et al,^63^ HSP70 is responsible for the initial folding of substrates and their loading into HSP90. Its affinity for unfolded substrates is tightly regulated by HSP40. Indeed, HSP40 not only delivers unfolded substrates to ATP-bound HSP70, but it also accelerates the hydrolysis of ATP, inducing a tighter binding of the substrate by HSP70. We speculate that MIR21 can interfere with this balance and thus, with the HSP90-mediated activation of client proteins, by modulating the expression of HSP40.

We have shown here that MIR21 drives resistance both in CCA and in non-CCA carcinoma cells. Thus, it is likely that these findings may be extended to a number of malignancies. Despite a general over-expression of MIR21 in cancer tissues, it is known that MIR21 is remarkably over-expressed in a proportion of cancer patients and may therefore serve as a valuable biomarker.^39^ In addition, there is evidence that levels of circulating MIR21 can define the prognosis of cancer patients and may act as surrogate for miRNA expression in the tumour.^34^ Thus, circulating MIR21 may represent an easily accessible tool for the identification of patients likely to benefit from treatment with HSP90 inhibitors.

Finally, we have provided initial evidence of the feasibility of developing human PDOs from CCA patients. To date, successful 3D organoids have been established from a variety of cancer types, but no evidence has been reported for biliary tract cancers. In these studies we show that PDOs could be derived from 1 biopsy core, indicating that this technology may be attractive for clinical implementation. Our studies indicate the possibility that PDOs may resemble the original tumor and may potentially be used for in vitro application and manipulation within 6–8 weeks from establishment. Thus, PDOs may represent a promising novel tool to guide treatment selection within the life expectancy of CCA patients, and may offer an additional platform that better recapitulates human cancers to investigate their biology.

## Supplementary Material

Supplementary Videos 1 and 2

## Footnotes

Note: To access the supplementary material accompanying this article, visit the online version of *Gastroenterology* at www.gastrojournal.org, and at https://doi.org/10.1053/j.gastro.2017.10.043.
