# Supplementary figures and images for: MIR21 Drives Resistance to Heat Shock Protein 90 Inhibition in Cholangiocarcinoma
Source: Gastroenterology. 2018 Mar;154(4):1066–1079.e5. doi: 10.1053/j.gastro.2017.10.043 (PMC5863695; doi:10.1053/j.gastro.2017.10.043)

## Slide 1
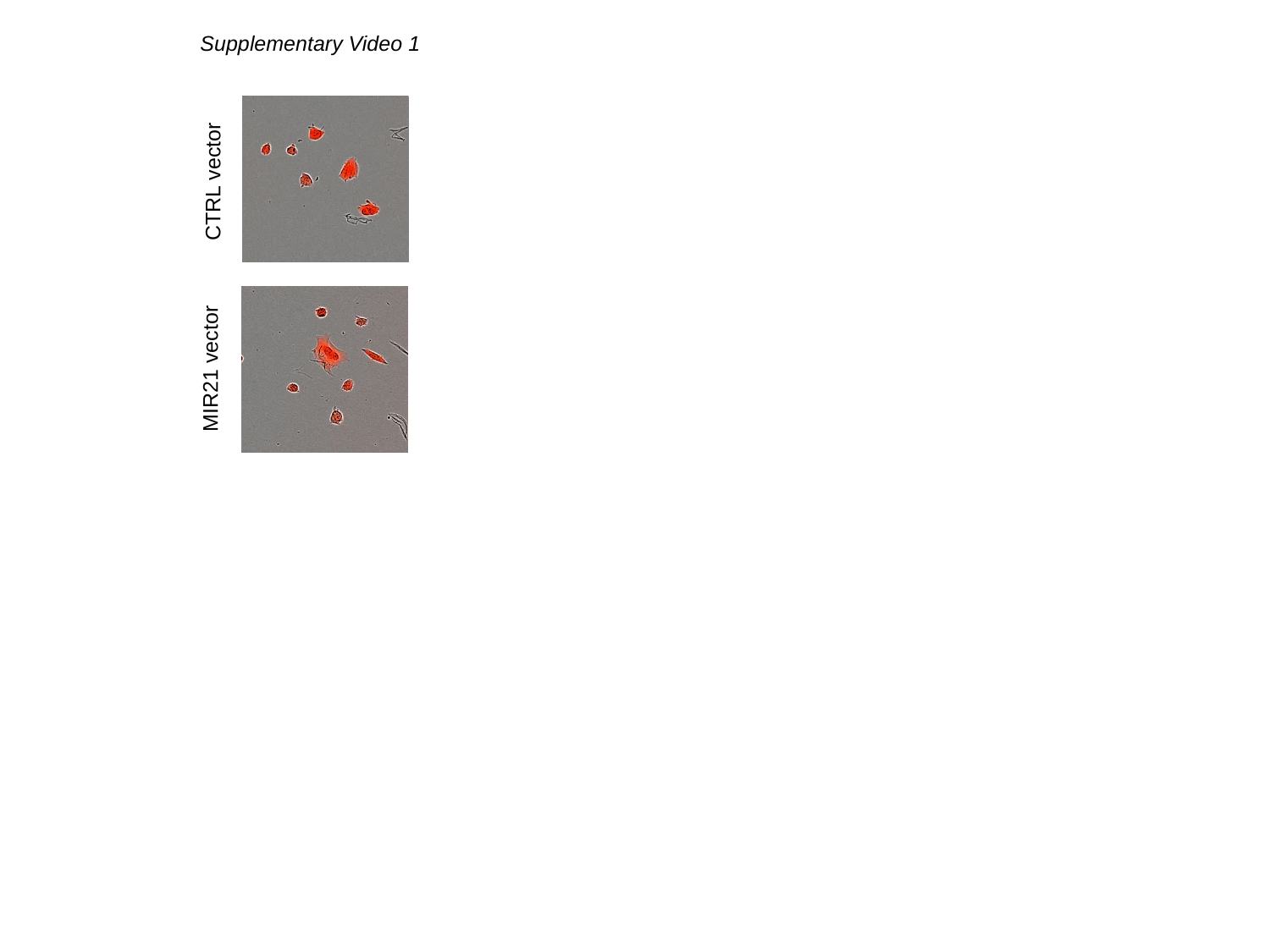

Supplementary Video 1
CTRL vector
MIR21 vector

## Slide 2
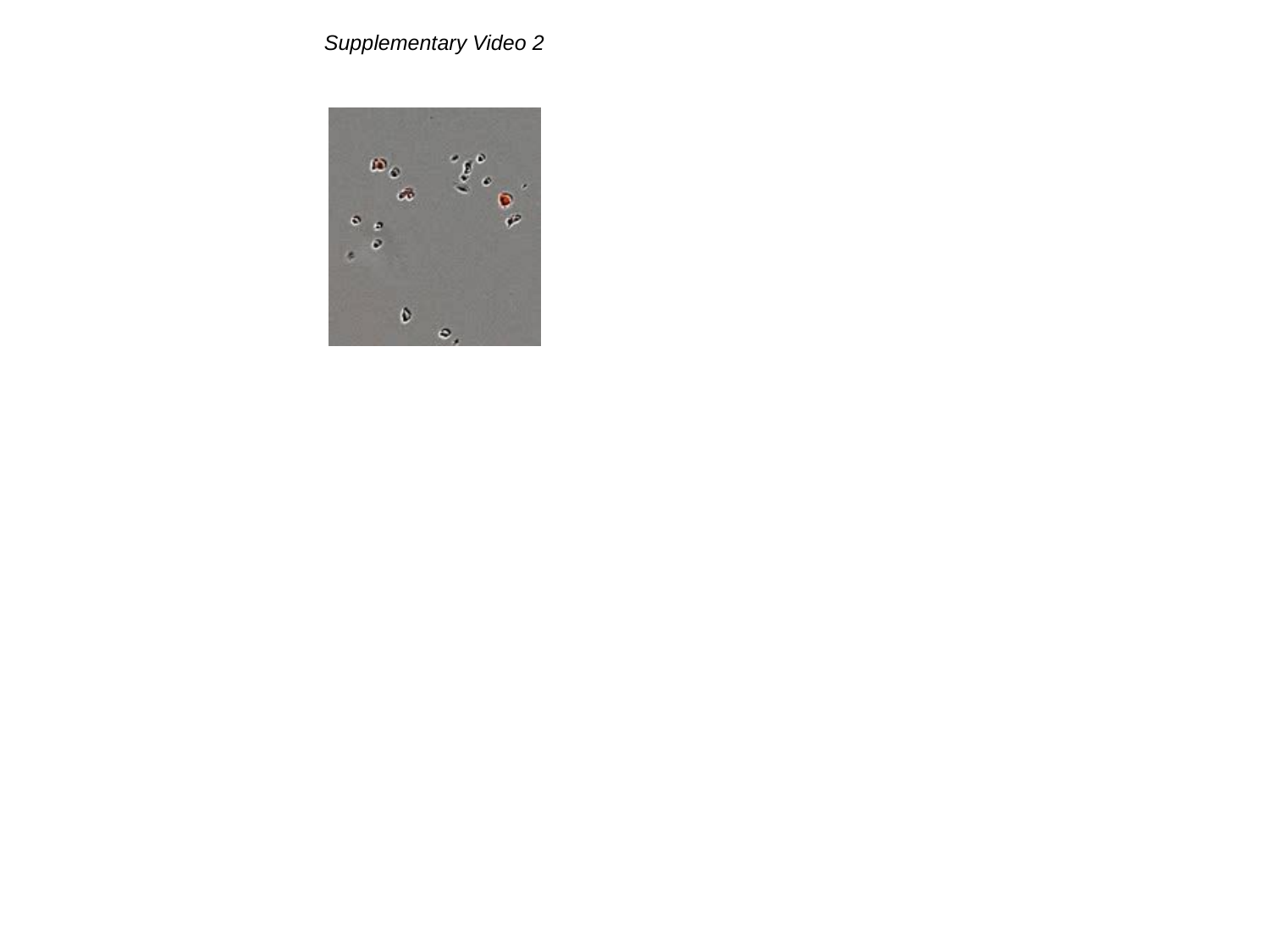

Supplementary Video 2

Supplement: Supplementary Videos 1 and 2 [file mmc1.pptx]
