# Supplementary material for: MIR21 Drives Resistance to Heat Shock Protein 90 Inhibition in Cholangiocarcinoma
Source: Gastroenterology. 2018 Mar;154(4):1066–1079.e5. doi: 10.1053/j.gastro.2017.10.043 (PMC5863695; doi:10.1053/j.gastro.2017.10.043)
Supplement: Supplementary Information [file mmc3.pdf]

SUPPLEMENTARY INFORMATION TO:

microRNA 21 Promotes Resistance of Cholangiocarcinomas to Heat Shock Protein 90 Inhibitors

Short title: miR-21 as biomarker of HSP90 inhibition

Andrea Lampis[1], Pietro Carotenuto[1], Georgios Vlachogiannis[1], Luciano Cascione[2], Somaieh Hedayat[1], Rosemary Burke[1], Paul Clarke[1], Else Bosma[1], Michele Simbolo[3], Aldo Scarpa[3], Sijia Yu[1], Rebecca Cole[1], Elizabeth Smyth[4], Javier Fernández Mateos[1], Ruwaida Begum[4], Blanka Hezelova[4], Zakaria Eltahir[4], Andrew Wotherspoon[4], Nicos Fotiadis[4], Maria Antonietta Bali[4], Chirag Nepal[5], Khurum Khan[5], Mark Stubbs[1], Jens C Hahne[1], Pierluigi Gasparini[6], Vincenza Guzzardo[7], Carlo M Croce[6], Suzanne Eccles[1], Matteo Fassan[3,7], David Cunningham[4], Jesper B Andersen[5], Paul Workman[1], Nicola Valeri[1,4], Chiara Braconi[1,4]

Supplementary methods

Cell lines. Intrahepatic (SW1, SNU-1079, CCLP), and extrahepatic (SNU-1196, TFK-1, EGI-1, SNU-245) CCA cell lines, along with gallbladder (WITT) and Ampulla of Vater (SNU-478) cancer cell lines, were purchased from the Leibniz Institute DSMZ-German Collection of Microorganisms and Cell Culture (Braunschweig, Germany), the Korean Cell Line Bank (Seoul, Korea) or were kindly provided by Prof. Stuart Forbes (University of Edinburgh). Cells were cultured in Dulbecco’s modified Eagle medium with 10 % foetal bovine serum. MIR21KO RKO colon carcinoma cells were purchased from Horizon Discovery (Cambridge, UK), while MIR21KO DLD-1 colon carcinoma cells were a kind gift from Jian Yu (University of Pittsburgh Cancer Institute) to Carlo Croce (Ohio State University). Cells were tested negative for Mycoplasma and authenticated through Short Tandem Repeat (STR) analysis.

Next generation sequencing of multiplex PCR amplicons. Two multigene panels were used: the 50-gene Ion AmpliSeq Cancer Hotspot panel v2 (Life Technologies, Paisley, UK) and an AmpliSeq custom panel targeting six genes not included in the commercial panel, as previously described[1]. The first explores selected regions of 50 cancer- genes: ABL1, AKT1, ALK, APC, ATM, BRAF, CDH1, CDKN2A, CSF1R, CTNNB1, EGFR, ERBB2, ERBB4, EZH2, FBXW7, FGFR1, FGFR2, FGFR3, FLT3, GNA11, GNAS, GNAQ, HNF1A, HRAS, IDH1, IDH2, JAK2, JAK3, KDR/VEGFR2, KIT, KRAS, MET, MLH1, MPL, NOTCH1, NPM1, NRAS, PDGFRA, PIK3CA, PTEN, PTPN11, RB1, RET, SMAD4, SMARCB1, SMO, SRC, STK11, TP53, VHL. The custom panel targets additional 6 genes: ARID1A, BAP1, PBRM1, PIK3C2A, PIK3C2G, TGFBR2. Twenty 20 ng of DNA was used for each multiplex PCR amplification. Emulsion PCR was performed with the OneTouch2 system (Life Technologies, Paisley, UK). The quality of the libraries was evaluated by on-chip electrophoresis in an Agilent Technologies’ 2100 Bioanalyzer (Santa Clara, USA). Sequencing was run on an Ion Torrent Personal Genome Machine (Life Technologies, Paisley, UK) loaded with 316 (50-gene panel) or 318 chips (6-gene panel). Data analysis, including alignment to the hg19 human reference genome and variant calling, was done using the Torrent Suite Software v.3.6 (Life Technologies, Paisley, UK). Filtered variants were annotated using the SnpEff software v.3.1[2]. Alignments were visually verified with the Integrative Genomics Viewer v.2.2 [3, 4].

Bioinformatics. For all statistical analyses the R language environment was used (v. 3.0.1; www.r-project.org). Hierarchical clustering was performed on cell viability data using the Pearson correlation as metric and complete linkage as method. We determined significant associations ($p<0.05$) between a selected mutation and drug sensitivity by Chi-square test with the Yates correction factor.

Real time PCR. RNA was extracted using Trizol (Invitrogen, Carlsbad, USA). Reverse transcription was performed with Taqman microRNA reverse transcription kit (Life Technologies, Paisley, UK), and miRNA expression assessed by qPCR with Taqman assay and normalized to that of RNU48 (Life Technologies, Paisley, UK)

Transfection. Cells were reversed transfected in 96-well plates using HiPerFect Transfection Reagent Qiagen, Hilden, Germany). For transient inhibition of miR-21 a locked nucleic acid (LNA™) miR-21 inhibitor or the Negative Control A LNA were used (Exiqon, Vedbaek, Denmark). In rescue experiments cells were transfected with DNAJB5-pCMV6 or Empty-pCMV6 (Origene, Rockville, MD, USA).

Cell viability. Cell viability was measured by CellTiter-Blue® Assay (Promega, Madison, WI, USA) and the GI_{50} derived using Prism Software (Graphpad, La Jolla, USA). For RFP+ cells cell viability was assessed by Incucyte Zoom live cell imaging (Essen Bioscience, Hertfordshire, UK) or by Celigo S (Nexcelom, Manchester, UK).

Live cell imaging. Cells were plated in 96-well plates and monitored with IncuCyte Zoom (Essen Bioscience, Hertfordshire, UK). Phase-contrast images and RFP+ images were taken every 4 hours.

Tetracycline-activated (Tet-on) inducible stable clones. The precursor miR-21 sequence was cloned into a TRIPZ lentivector (Dharmacon, Little Chalfont, UK) using PCR amplification of target region and digestion with Cla I and Mlu I restriction enzymes (New England Biolabs, Ipswich, Massachusetts, USA). Packaging of viral particles and target cell lines

infections were performed using HEK293T. Overexpression of miR-21 was confirmed by Taqman assay. Plasmid with antimiR-21 sequence was produced by amplification and cloning of antimiR-21 sequence, with standard cloning procedures, from miRZip^{TM}-miR-21 plasmid (System Bioscience, Palo Alto, CA, USA) in order to generate an equivalent inducible system for silencing miR-21 expression. Cell growth optimization for plating density has been performed for cells with miR-21 manipulation to take into consideration the effect of miR-21 on cell growth.

Human heat shock protein antibody array. Cells were solubilized in 1X lysis buffer containing a protease inhibitor cocktail. Membrane antibody arrays (RayBiotech, Norcross, USA) were blocked with 1 mL of blocking buffer for 30 min. 500 µg of extracted proteins were diluted in 1 mL of blocking buffer and dispersed on top of membrane antibody arrays overnight at 4 °C. Detection was performed as per the manufacturer's instructions and the signal measured using Licor system (Licor, Lincoln, NE, USA).

Western blot. Immunoblotting was performed as previously described[5]. Incubation with primary antibodies for DNAJB5 (ab101514, Rabbit; Abcam, 1:1000 dilution), HSP70 (ab182844, Rabbit, Abcam; 1:5000 dilution), Beta-Actin (Anti-Actin, Clone C4, Mouse, MP Biomedicals; 1:10000 dilution) was performed overnight at 4°C. Secondary HRP-conjugated polyclonal Goat Anti-Rabbit or Goat Anti-Mouse antibodies (Cell Signaling, Danvers, MA, USA;1:10000) were used. Prime ECL (Amersham, GE healthcare) was used to develop signal as manufacturer's instructions with Licor imaging system.

Luciferase assays. Cells were transfected with 1 µg DNAJB5-pMirTarget or pMiRTarget CTRL (Origene, Rockville, USA) with HiPerFect Transfection Reagent (Qiagen, Hilden,

Germany) and the luciferase activity measured after 48 h using the Dual Glo Assay system (Promega, Madison, WI, USA) according to the manufacturer's protocol in a multiwell plate luminometer (Perkin-Elmer, Seer Green, Beaconsfield, UK). Luciferase activity was normalized to that of renilla activity for each transfected well.

Immunohistochemistry**:** Immunohistochemical stains were automatically performed in 3-4 μm sections using the Bond Polymer Refine Detection kit (Leica Biosystems, Newcastle upon Tyne, UK) in the BOND-MAX system (Leica Biosystems), according to the manufacturer’s specifications. Appropriate positive and negative controls were run concurrently. The following antibodies were used: DAKO mouse monoclonal CK7 (Agilent, Santa Clara, CA, USA), DAKO mouse monoclonal CK19 (Agilent, Santa Clara, CA, USA), rabbit polyclonal DNAJB5 (Sigma-Aldrich, Haverhill, UK) and rabbit polyclonal HSP70 (Abcam, Cambridge, UK). DNAJ5B was classified according to a 4-tiered scoring system based on the intensity of protein expression as follows: 0: indicates no stain or stain in less than 10% of tumour cells; 1+: faint/weak cytoplasm/nuclear stain in 10% or more of cells; 2+: moderate cytoplasm/nuclear stain in 10% or more of tumour cells; and 3+: strong cytoplasm/nuclear stain in 10% or more of tumour cells.

***In Situ* RNA hybridization.** A locked nucleic acid (LNA) probe with complementarity to a 21-bp section of miR-21 was labelled with 5′-digoxigenin and synthesized by Exiqon. Tissue sections were digested with ISH protease 1 (Ventana Medical Systems) and *in situ* hybridization performed as described [6]. Negative controls included omission of the probe and the use of a scrambled LNA probe. Each sample was classified according to a 4-tiered scoring system based on the intensity of miR-21 expression as follows: 0: indicates no stain or stain in less than 10% of tumour cells; 1+: faint/weak cytoplasm/nuclear stain in 10% or

more of cells; 2+: moderate cytoplasm/nuclear stain in 10% or more of tumour cells; and 3+: strong cytoplasm/nuclear stain in 10% or more of tumour cells. In all the considered tissue samples, fibroblasts featured miR-21 expression and were assumed as positive internal control (not considered in ISH score).

Supporting Table legend

Supporting Table 1. List of drugs included in the library used for the HTS.

Supporting Table 2. Comparison between mutations found in human CCA tissues and human CCA cell lines using the same NGS gene-panel. Mutations that were found to be present in >10% of human tissue are shown in the left column [1]; mutations that are represented in our cell lines are depicted with an "x".

Supporting Table 3. List of drugs that were statistically significantly ($p<0.05$) active at all the 3 concentration in each cell line. Compounds that are commonly represented across all the iCCA or the eCCA cell lines are depicted in yellow and light blue respectively.

Supporting Table S4. Statistical analysis of experiments in Fig 2E. P value indicates unpaired two-tailed ttest. Fold changes (FC) in cell viability are reported for day 3 that represents the timepoint at which the assessment of response was performed following miR-21 over-expression.

Supporting Table 5. Statistical analysis of animal experiments. P value indicates unpaired two-tailed ttest.

Supporting video legends

Video 1. miR-21KO DLD1 cells were stably infected with an inducible viral vector over-expressing miR-21 or CTRL. Cells were exposed to doxycycline (1μg/ml) to activate miR-21/CTRL and RFP expression that were under the same promoter. RFP+ (red) cells represented activated infected cells. Activated cells were plated in 96-well plates and AUY-922 added 22 hrs later. Cell viability and RFP confluency were monitored and measured at interval periods through the Incucyte Zoom. Data represent mean and STDEV of 12 replicates.

Video 2. miR-21KO DLD1 cells were co-cultured with Tet-on miR-21KO miR-21 vector DLD-1 cells. Cells were activated, plated in 96-well plate, exposed to doxycycline, and treated with AUY-922. RFP+ cells represented cells that over-expressed miR-21, while RFP-(bright-field) cells represented miR-21KO cells.

Supplementary references

1. Simbolo M, Fassan M, Ruzzenente A, et al. Multigene mutational profiling of cholangiocarcinomas identifies actionable molecular subgroups. Oncotarget 2014;5:2839-52.
2. Cingolani P, Platts A, Wang le L, et al. A program for annotating and predicting the effects of single nucleotide polymorphisms, SnpEff: SNPs in the genome of Drosophila melanogaster strain w1118; iso-2; iso-3. Fly (Austin) 2012;6:80-92.
3. Robinson JT, Thorvaldsdottir H, Winckler W, et al. Integrative genomics viewer. Nat Biotechnol 2011;29:24-6.
4. Thorvaldsdottir H, Robinson JT, Mesirov JP. Integrative Genomics Viewer (IGV): high-performance genomics data visualization and exploration. Brief Bioinform 2013;14:178-92.
5. Carotenuto P, Fassan M, Pandolfo R, et al. Wnt signalling modulates transcribed-ultraconserved regions in hepatobiliary cancers. Gut 2017;66:1268-1277.
6. Braconi C, Valeri N, Kogure T, et al. Expression and functional role of a transcribed noncoding RNA with an ultraconserved element in hepatocellular carcinoma. Proc Natl Acad Sci U S A 2011;108:786-91.
